# Preoperative estimation of distance between retinal break and limbus with wide-field fundus imaging: Potential clinical utility for conventional scleral buckling

**DOI:** 10.1371/journal.pone.0212284

**Published:** 2019-02-12

**Authors:** Keijiro Ishikawa, Ri-ichiro Kohno, Eiichi Hasegawa, Shintaro Nakao, Shigeo Yoshida, Koh-Hei Sonoda

**Affiliations:** 1 Department of Ophthalmology, Graduate School of Medical Sciences, Kyushu University, Fukuoka, Japan; 2 Department of Ophthalmology, Kurume University School of Medicine, Kurume, Japan; Massachusetts Eye & Ear Infirmary, Harvard Medical School, UNITED STATES

## Abstract

**Objective:**

Accurate scleral marking of retinal breaks is essential for successful scleral buckling. This study aimed to investigate the use of wide-field fundus images obtained with an Optos for preoperative estimation of the distance from the limbus to the retinal breaks.

**Methods and analysis:**

This is a retrospective review of 29 eyes from 26 patients with rhegmatogenous retinal detachment who received scleral buckling with anatomically successful repair. They underwent wide-field fundus photography with Optos California. In the pre- and postoperative fundus images, we measured distances from the macula to the retinal tears (T_M_), to the center of the vortex veins (V_M_), to the optic disc (D_M_), and to the posterior edge of the scleral buckle (B_M_).

**Results:**

(BM—V_M_) / D_M_ was significantly correlated with the distance from the limbus to the posterior edge of the scleral buckle that had been determined intraoperatively. (*r* = 0.705; p<0.001) We applied a regression line derived from this correlation with the value of (T_M_ -V_M_) / D_M_ in order to calculate estimated distances between retinal breaks and the limbus. The calculated distances were all within the range of distances from the limbus to the anterior and posterior edges of the scleral buckles.

**Conclusion:**

Preoperative analysis of Optos images may be useful for estimating the distance from the limbus to retinal breaks, which might aid scleral marking during scleral buckling surgery.

## Introduction

Recent advances in vitrectomy instrumentation and techniques have expanded its application to the repair of rhegmatogenous retinal detachment (RRD). However, scleral buckling is still preferred in particular cases, such as those with RRD caused by breaks in lattice degeneration without posterior vitreous detachment.

Accurate scleral marking of retinal breaks with scleral indentation during observation is essential for successful scleral buckling. Many recent studies have demonstrated the efficacy of a non-contact wide-angle viewing system (WAVS) combined with chandelier endoillumination during the scleral buckling procedure. [1–13] This WAVS-assisted scleral buckling has advantages over the conventional indirect ophthalmoscopy-guided technique in observing retinal tears and the subsequent scleral marking. However, a very recent case report has raised a potential concern regarding infectious endophthalmitis development with the use of a chandelier endoilluminator during scleral buckling. Although the conventional indirect ophthalmoscopy-guided technique requires considerable surgical skill, mastery of the skill is still important for clinicians since its long-term safety has been proven with low incidence of endophthalmitis after conventional scleral buckling. [14]

The Optos Ultra-Widefield Fundus Camera (Optos, Dumfermline, UK) has proven useful in documenting the preoperative state of RRD with acquisition of 200° panoramic images of the retina. [15] In the present study, we investigated whether the preoperative Optos image in RRD cases can facilitate estimation of scleral chord length from the limbus to the retinal tears, which might aid scleral marking during scleral buckling surgery.

## Materials and methods

This was a retrospective, noncomparative case series. This study was approved by the Institutional Ethics Committees of the Kyushu University Hospital (Protocol No. 29304, UMIN000031945), and was performed in accordance with the ethical standards laid down by the Declaration of Helsinki. All data collection conformed to the Declaration of Helsinki for research involving human subjects.

### Subjects

The subjects included patients whose fundus images that were taken with the method described in Image Acquisition allowed identification of retinal breaks, and who underwent explant scleral buckle surgery using silicone sponge primarily for rhegmatogenous retinal detachment, with anatomically successful repair between January 2016 and June 2017 at the Department of Ophthalmology, Kyushu University Hospital. Patients who required simultaneous cataract surgery and/or pars plana vitrectomy were excluded. The total number of eyes included for analysis was 29 eyes of 26 patients.

Axial length (AL) was measured preoperatively with a Zeiss IOL-Master laser interferometer (Optical Biometry, IOL Master; Carl Zeiss Meditec AG, Jena, Germany). Based on the AL, the 29 eyes were divided into two groups, non-highly myopic patients (group A, AL<26 mm) and highly myopic patients (group B, AL≧26 mm). [16]

### Image acquisition

Color fundus images were taken using the Optos California (Dunfermline, Scotland, UK). Macula-centered ultra-widefield color fundus images and those obtained in the four steering positions (left, right, up and down) from each eye were obtained one day before and two to four days after scleral buckle surgery. The images were taken when the “green in-focus” signal was obtained.

### Data collection and image analysis

**Fig 1:**
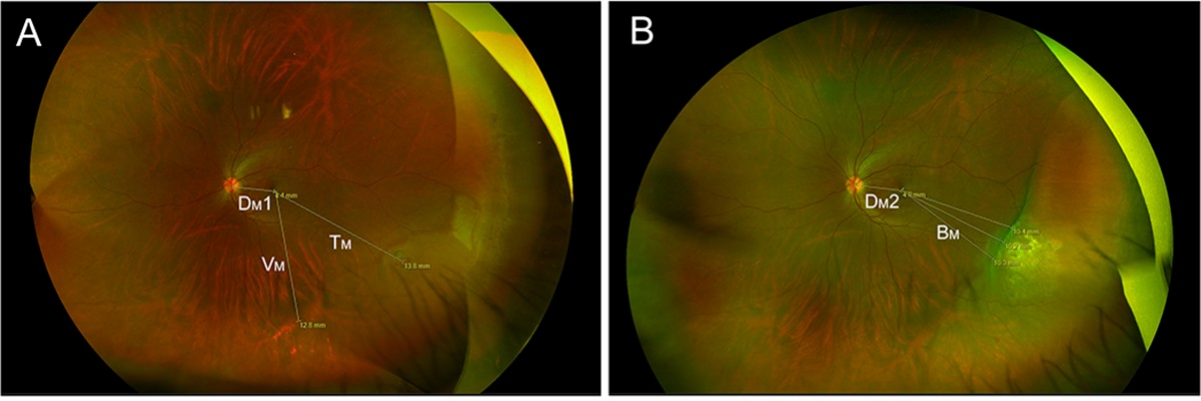
Image analysis of preoperative and postoperative Optos images of an eye with rhegmatogenous retinal detachment. (A) In the preoperative ultra-widefield fundus image of an eye with rhegmatogenous retinal detachment, distances from the fovea to the retinal tear (T_M_), to the center of the vortex vein ampulla (V_M_) and to the center of the optic disc (D_M_1) were measured. (B) In the postoperative fundus image of the eye, distances from the fovea to the posterior edge of scleral buckle (B_M_) and to the center of the optic disc (D_M_2) were measured.

The Optomap auto-montage software (Dunfermline, Scotland, UK) can produce simultaneous views of the central pole, mid-periphery and periphery up to 97% or 220° of the retina from the images taken in different positions using the multi-capture, montaging functionality. In addition, this software allows distance measurement in the images. In the wide-field fundus images obtained with the software, we determined the center of the disc and the center of the vortex vein ampulla located in the same quadrant as the retinal tears as described previously. [17] In the preoperative fundus images, we measured distances from the fovea to the center of retinal tears (T_M_), to the center of the vortex vein ampulla (V_M_), and to the center of the optic disc (D_M_1). In the postoperative fundus images, we measured distances from the fovea to the center of optic disc (D_M_2). The average distance from the fovea to the posterior edge of scleral buckle (B_M_) was calculated by averaging the distances measured from the fovea to three different buckle edges. A representative image taken from case number 19 (Table 1) illustrates these distance measurements in the wide-field fundus images obtained with the Optomap auto-montage software (Fig 1).

**Table 1:** Preoperative findings.

Case Number	Age, Years	Sex	Eye	Lens Status	Pre- operative AL (mm)	Pre-operative BCVA	Extent of RD, Quadrants	Location of Retinal breaks	Retinal Breaks Number	Evacuative SRF	Material of Buckle	Type of Buckle	Post- operative AL (mm)
1	63	F	R	P	24.04	1.2	1	Superonasal	1	N	Silicone sponge	Segmental	24.12
2	38	M	L	P	24.5	1.5	4	Inferotemporal	4	Y	Silicone sponge	Segmental	24.31
3	38	M	R	P	24.5	1.5	1	Superotemporal	1	N	Silicone sponge	Segmental	24.7
4	43	M	R	P	24.72	1	2	Superonasal	1	Y	Silicone sponge	Segmental	24.76
5	41	M	L	P	24.84	0.4	5	Inferior	2	Y	Silicone sponge	Segmental	25.02
6	21	M	L	P	24.9	0.9	4	Inferior	4	Y	Silicone sponge	Segmental	25.17
7	41	M	R	P	24.9	1.2	5	Inferior	4	Y	Silicone sponge	Segmental	25.8
8	40	F	L	P	25.46	0.6	3	Inferotemporal	1	Y	Silicone tire	Segmental	25.6
											+ Silicone band	+Encircling	
9	15	M	R	P	25.71	1.2	9	Superotemporal	1	N	Silicone sponge	Segmental	25.26
10	24	F	L	P	25.74	1.2	2	Inferior	3	N	Siliconesponge	Segmental	26.12
											+Silicone band	+Encircling	
11	23	M	L	P	25.97	1.2	3	Inferotemporal	1	N	Silicone sponge	Segmental	25.52
12	23	F	R	P	26.1	1.2	3	Inferonasal	1	Y	Silicone sponge	Segmental	25.84
13	47	F	R	P	26.1	1	2	Superonasal	1	N	Silicone sponge	Segmental	26.02
14	23	M	R	P	26.1	1.2	2	Superotemporal	3	N	Silicone sponge	Segmental	25.98
15	28	F	R	P	26.5	1.2	3	Inferotemporal	6	Y	Silicone sponge	Segmental	26.27
16	40	M	L	P	26.78	1.2	3	Superotemporal	2	Y	Silicone sponge	Segmental	26.68
17	28	M	L	P	26.86	1.5	6	Superotemporal	4	Y	Silicone sponge	Segmental	26.89
18	38	M	R	P	26.86	1.2	6	Superotemporal	1	Y	Silicone sponge	Segmental	26.94
19	35	M	L	P	26.9	1.2	1	Inferotemporal	1	N	Silicone sponge	Segmental	26.76
20	28	M	R	P	27.38	0.5	5	Inferotemporal	4	Y	Silicone sponge	Segmental	26.92
21	42	F	L	P	27.44	1.2	5	Superotemporal	2	N	Silicone sponge	Segmental	27.52
22	31	F	L	P	27.50	0.4	2	Inferotemporal	1	N	Silicone sponge	Segmental	27.68
23	43	M	R	P	27.59	1.2	4	Inferotemporal	3	Y	Silicone sponge	Segmental	27.55
24	17	M	R	P	28.08	0.2	10	Superonasal	4	Y	Silicone sponge	Segmental	27.52
25	43	F	R	P	28.41	1.2	2	Inferotemporal	3	N	Silicone sponge	Segmental	28.52
26	47	M	R	P	28.41	1.2	2	Superotemporal	1	Y	Silicone sponge	Segmental	28.67
27	33	F	L	P	28.49	1.2	3	Inferotemporal	2	N	Silicone sponge	Segmental	28.45
28	46	M	R	P	28.51	1.2	4	Superior	6	Y	Silicone sponge	Segmental	28.48
29	44	M	L	P	29.11	1.2	3	Superior	2	Y	Silicone sponge	Segmental	29.08

AL, axial length. BCVA, best-corrected visual acuity. RD, retinal detachment. SRF, subretinal fluid. P, phakic.Y, yes. N, no.

From the medical records of each case, we collected surgical information including the scleral chord lengths from the limbus to the anterior and posterior edges of the placed buckles that were measured intraoperatively by caliper after buckle placement by the surgeons.

To test the correlation of the length difference between B_M_ and V_M_ and the location of the buckle’s posterior edge that was determined intraoperatively, correlation analysis was conducted to examine the relationship between the value of posterior buckle edge position toward the vortex vein ampulla, which was calculated as B_M_/D_M_2-V_M_/D_M_1, and the scleral chord length from the limbus to the posterior buckle edge (SCL1).

A regression line with SCL1 as the dependent variable (y-axis) and B_M_/D_M_2-V_M_/D_M_1 (as predictor) was derived, and the correlation coefficient (Pearson’s) was calculated. A p value <0.05 was considered to indicate significant differences. Statistical analyses were performed using JMP Pro 13.0 (SAS Institute, Cary, NC, USA).

## Results

### Patient demographics

The data and images were obtained from 29 eyes of 26 patients (16 men and 10 women; average age, 35.3 ± 10.7 years; range, 15–63 years), including 11 eyes with AL<26 mm (group A) and 18 eyes with AL≧26 mm (group B). All patients underwent explant scleral buckling surgery using conventional indirect ophthalmoscopy with anatomically successful repair. The mean AL of all eyes was 26.5 mm. The mean preoperative best-corrected visual acuity (BCVA) was 0.003 ± 0.199 LogMAR, ranging from 0.70 to -0.18 LogMAR. All eyes were phakic. Preoperative characteristics of the cases and eyes are summarized in Table 1.

### Correlation between intraoperative and postoperative Optos image-based determination of the buckle’s posterior edge location

**Fig 2:**
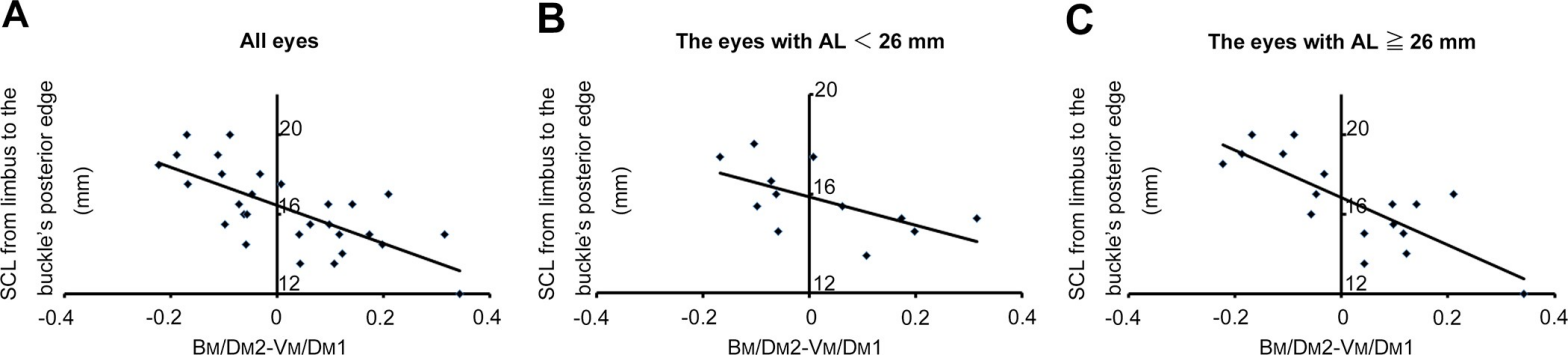
Correlation between intraoperative measurement of scleral chord length from limbus to posterior edge of buckle and estimated measurement derived from postoperative Optos imaging. (A) Correlation between B_M_/D_M_2-V_M_/D_M_1(B-V) and the scleral chord length (SCL) from the limbus to the posterior buckle edge (*r* = 0.595; p<0.001, all eyes, n = 29). (B) In the analysis of eyes with axial length (AL) less than 26 mm, SCL = -2.8484 × B-V + 15.893; *r* = 0.602; p = 0.038, n = 11). (C) In the analysis of eyes with AL of 26 mm or more, SCL = -5.9636 × B-V + 16.822; *r* = 0.786; p<0.001, n = 18).

First, to investigate whether analysis of object location in the peripheral retinas in Optos fundus images can predict the scleral chord length from the limbus to the objects, we assessed the correlation between the value of B_M_/D_M_2—V_M_/D_M_1 (B-V) and the scleral chord length from the limbus to the posterior buckle edge (SCL1). In combined group analysis, the values of B-V were significantly correlated with SCL1 (*r* = 0.705; p<0.001). There was a good correlation between B-V and SCL1 in subgroup analysis (Group A: SCL1 = -2.8484 × B-V + 15.893; *r* = 0.602; p = 0.038, n = 11, Group B: SCL1 = -5.9636 × B-V + 16.822; *r* = 0.786; p<0.001, n = 18) (Fig 2).

These data demonstrate that scleral chord length from the limbus to the buckle’s posterior edge is correlated with the distance between the buckle’s posterior edge and the vortex vein ampulla that is calculated from the postoperative Optos fundus image. A regression line derived from these correlations was applied to the following calculation of estimated scleral chord length from the limbus to retinal breaks based on preoperative analysis of Optos fundus images.

### Calculation of estimated scleral chord length from limbus to retinal breaks

**Fig 3:**
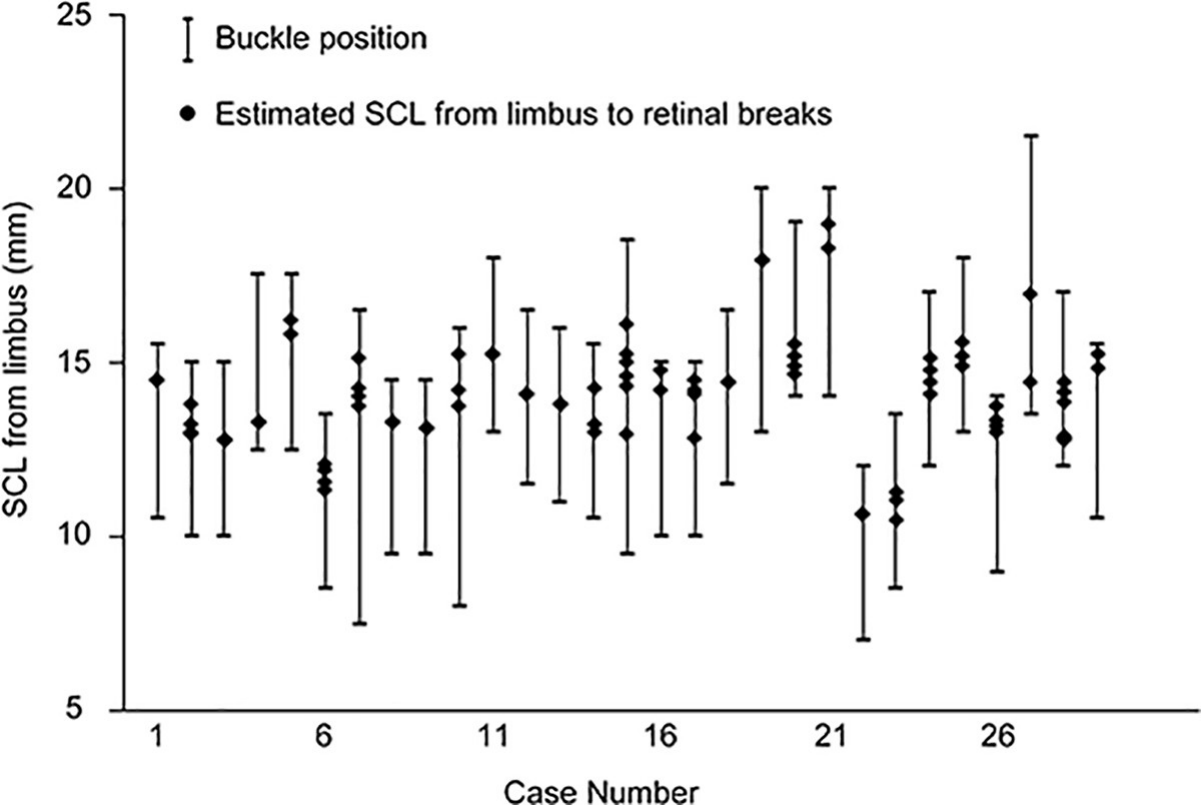
Graph comparing estimated scleral chord lengths based on preoperative Optos imaging with intraoperative measurements of scleral chord length. Estimated scleral chord length(s) (SCL) from the limbus to retinal breaks (●) was (were) calculated by applying the regression line with the value of (T_M_-V_M_)/D_M_1 in each case. The bars indicate the buckle positions that range between the distances from the limbus to the anterior buckle edge and those from the limbus to the posterior buckle edge.

Next, we applied the regression line derived from the correlation with (T_M_-V_M_)/D_M_1 in order to calculate the estimated scleral chord length from the limbus to the retinal breaks. The estimated scleral chord lengths from the limbus to the retinal breaks were calculated using -2.8484 × (T_M_-V_M_) / D_M_1 + 15.893 for group A(Case Number 1 to 11), and -5.9636 × (T_M_-V_M_) / D_M_1 in + 16.822 for group B (Case Number 12 to 29). The calculated distances (black dots) were all within the range of distances from the limbus to the anterior and posterior edges of scleral buckles (bars) that were measured during surgery and collected from medical records (Fig 3).

In the eyes with axial length (AL) less than 26 mm (Case Number 1 to11), estimated SCLs were calculated using -2.8484 × (T_M_-V_M_)/D_M_1 + 15.893. In the eyes with AL of 26 mm or more, (Case Number 12 to 29), estimated SCLs were calculated using -5.9636 × (T_M_-V_M_)/D_M_1 + 16.822.

These results suggest that preoperative calculation of estimated scleral chord lengths from limbus to the retinal breaks can approximate the actual scleral chord lengths from the limbus to the posterior and anterior buckle edges.

## Discussion

This study demonstrates that preoperative Optos image-based analysis may facilitate scleral chord length estimation from the limbus to the retinal tears, which might be useful for determining the location of scleral buckle placement during scleral buckling surgery.

Since the Optos creates planar images from the spheric fundus, there is magnification that prevents accurate distance measurement in the fundus [18, 19], however, a new Optomap software enables anatomically correct measurements on Optos fundus images.

There is at least one vortex vein ampulla per quadrant at the equator [20] and retinal tears are usually found around the equator or in the peripheral retina defined as the area between the vortex vein ampullae and the ora serrata. Thus, we set the vortex vein ampullae as a reference point in the fundus to measure the distance of the objects in the area. Indeed, the distance from the buckle’s posterior edge in the postoperative fundus images were significantly correlated with those from the limbus that were measured during surgery. These results suggest that our measurement setting is applicable for prediction of the SCL from the limbus based on Optos image analysis.

As regression models derived from the SCLs of retinal tears from the limbus and Optos image analysis are useful for direct prediction of their locations on the sclera, the SCLs of retinal tears measured during surgery should therefore be used in our analyses. However, we were unable to identify all the SCLs of retinal tears from the limbus from the medical records of the reviewed cases. This is presumably because accurate measurement of SCLs of retinal tears from the limbus during surgery is technically difficult since it requires the precise scleral marking that are perfectly located on the retinal tears. Thus, we utilized the SCLs from the limbus to the posterior buckle edge that were measured intraoperatively after buckle placement.

Theoretically, the actual length of a distance measured in the Optos image can be longer in larger eyes compared to smaller eyes. In fact, the Optomap software calculates the length equivalent to the setting of an eye with an AL of 24mm, and thus the calculated distance should be inaccurate in eyes with different axial lengths. To compensate for the inaccuracy of the measurement, we applied the ratio to D_M_ as the value but not for the calculated distance for the following calculation. In addition, we acquired different regression lines separately from highly myopic and non-highly myopic eyes and applied those to the subsequent calculation of the estimated scleral chord length. The values of the y-intercept in the regression lines, which were derived from the correlation analysis of the buckle’s posterior edge location, correspond to the average scleral chord length from the limbus to the vortex vein ampulla. The values of the y-intercept derived from the correlation analysis were 15.893 and 16.822 in group A and B, respectively. Our higher value in eyes with a longer axis indicate a longer scleral chord length from the limbus to the vortex vein ampulla in these eyes, which is consistent with previous findings from autopsy eyes showing correlation of ocular diameters and the distance between vortex exit sites and the limbus. [21]

There was far greater interpatient variability in buckle positioning than in the estimated SCLs from the limbus to the retinal breaks, the majority of which ranged between 11mm and 16mm (Fig 3). Retinal breaks are mostly located around the equatorial region and its peripheral area, especially in RRD cases of young patients to whom scleral buckling procedures are preferably applied. [22] In contrast, buckle positioning is determined according to the surgeons’ decisions made in each RRD case. Specifically, in RRD cases associated with horseshoe retinal tears, more coverage of the periphery rather than the tear position is intentional to release the vitreous traction in the periphery. The greater variability of buckle position can originate from the variability in the surgeons’ decision making in surgical strategies against RRD.

Limitations of our current study include its retrospective design and relatively small sample size. To evaluate the accuracy of the estimated scleral chord lengths based on preoperative Optos image analyses, it is necessary to design a prospective study comparing preoperative estimated length and the actual scleral chord length from the limbus to the retinal tear acquired by measuring distances between the limbus and a scleral marking during surgery. Another limitation is that our methodology is unsuitable for cases with bullous rhegmatogenous retinal detachment where a large amount of subretinal fluid and high retinal protrusion is present. In such cases, the estimated scleral chord lengths from the limbus to retinal tears based on the analyses of preoperative fundus images should theoretically be shorter than the actual scleral chord lengths of tear locations on the sclera. However, our present study did not include such bullous retinal detachment cases, which were treated with vitrectomy procedures rather than with scleral buckling. Recently, vitrectomy procedures have been preferred for treatment of cases with bullous retinal detachment, which are usually accompanied by vitreous liquefaction and/or posterior vitreous detachment as release of vitreous traction with vitrectomy is required for successful retinal reattachment in such cases.

With the expanding application of vitrectomy surgery for RRD repair, opportunities for performing scleral buckling surgery is reduced. Consequently, physicians-in-training would have greater difficulty in acquiring mastery of surgical procedures involved in scleral buckling, including marking of the retinal breaks on the sclera while observing the fundus, with subsequent positioning and suturing of the buckling material to the sclera. [2] Preoperative estimation of the retinal break location in the sclera can facilitate greater ease and accuracy of the scleral marking procedure, which may contribute to shorter surgery time and higher success rate of retinal reattachment with conventional scleral buckling.
